# Observations on Dr. Bellamy's Case of Lacerated Urethra

**Published:** 1809-06

**Authors:** Samuel Spencer

**Affiliations:** Duffield


### *Observations on Dr. Bellamy's Case of lacerated Urethra.*

I confess I have but little pretensions to animadvert on the practice of so experienced a surgeon as Dr. Bellamy. The case of lacerated urethra, which he has related to us in the Medical Journal for March last, is highly interesting, and, as he well observes, "full of practical importance;" and as it is one, moreover, that may occur to almost any practitioner, however remotely situated, it becomes desirable that we should have some settled ground to go upon. I would ask, therefore, whether the catheter ought generally to be had recourse to, as a part of the treatment of such a case? Much less, as it appears to me, could its mere introduction be required, while both the surgeon and the patient were satisfied that the bladder was regularly emptied, and remained "flaccid" within the pelvis; "as if," to use the words of Dr. Bellamy, "there was not a drop of water in it." Secondly, might not a more prompt and decided incision in the perineum have retarded at least, if not obviated, the catastrophe which ensued? For what gave fatality to the case but the lodgment of accumulated urine? To proceed. In justice to Dr. Bellamy, whose candour deserves every praise, it must be observed, that punctures were made in the scrotum, with the intention of evacuating the effused urine; but they only, by this "oozing," "just kept the swelling at bay;" they did not unload the perineum, which yet remained "very full and hard;" and even under their operation, symptoms of a bad aspect kept rapidly advancing. At length an incision was made in the perineum, and "seeing the great good of this, a still more ample one was made on the other side." Every reader of the case, I think, must lament, in common with myself, that this operation should have been so long deferred, under the delusive hope of matter forming. Even in this stage of the case, however, when the symptoms but too clearly evinced the near approach of gangrene, we are told of the great good which it effected; doubtless, a more early adoption of it would have proved an effectual barrier to the bad tendency of the case, but it could not arrest the progress of mortification.

Dr.

Dr. Bellamy, in my mind, seems to have depended too much on the rigors that succeeded to this severe accident, from whence he was induced hourly to expect an abscess in the perineum, which truly might have brought the case to a more favorable issue. So persuaded indeed was he of this happening, that after very justly despairing of the case, the occurrence of a fresh rigor renews his hopes, and he again looks forwards to the "formation of matter." —But these violent and renewed agitations of the body might occur without an impending abscess; in the instance before us, they seem to have been peculiarly connected with the escape of the urine from its proper channel, the sense of which was so painful to the patient, as to induce him to "cry out bitterly for a few moments." Were not the rigors, so distinguished in this case, akin to those anomalous shakings which sometimes attend the progress of a gall-stone through the biliary duct? The introduction of a catheter, we are told, in some habits, is followed by a like effect; and further, the application of a caustic to a stricture in the urethra "sometimes makes the most vigorous and hardy man tremble and shiver for ten minutes, with the pale and contracted visage of one in terror." We do not therefore, on every such occurrence, invariably look to the formation of matter. By the sectio cadaveris, scarce a "layer of matter" could be found. The tumour in the groin even, the extreme sensibility, redness, and distinct prominency of which, so strongly indicative of abscess, was found to contain only effused urine; which the overcharged perineum could not admit.

A delay in sending up this Paper, has given me an opportunity of noticing the case of contused urethra by Verax, in your last Number; in which we have an example strongly enforcing the opinion above inculcated: namely, that nothing in accidents of this sort can serve the patient but a prompt incision in the perineum; and, that effected, a most distressing and formidable disease becomes perfectly simple and manageable. Allow me to observe, that no Author with which I am acquainted, treats of this accident so distinctly and ably as Mr. John Bell, in the second Volume of his Principles of Surgery. Indeed, it will be obvious, that these Observations arise principally out of that elaborate performance.

I am, &c.

SAMUEL SPENCER.

